# Acceleration and Injury-Frame Biomechanics in Achilles Tendon Rupture: A Systematic Review

**DOI:** 10.3390/jfmk11030309

**Published:** 2026-08-08

**Authors:** Robert Olivar, Eleftherios Kellis

**Affiliations:** Laboratory of Neuromechanics, Department of Physical Education and Sport Sciences at Serres, Aristotle University of Thessaloniki, 62100 Serres, Greece; rolivar@phed-sr.auth.gr

**Keywords:** Achilles tendon rupture, video analysis, biomechanics, kinematics, false step, systematic review, NBA

## Abstract

**Background and Objectives**: Achilles tendon rupture (ATR) incidence has increased across sport populations, and, given its impact on elite performance, the purpose of this systematic review was to synthesize the available video analysis evidence describing the biomechanics of ATR in sport. **Methods**: Four databases were searched to June 2026 for video analysis studies reporting the mechanics of ATR in athletes. Eleven studies (2019–2026) reporting overlapping samples of ATR events in professional basketball, soccer, and American football were included and synthesized at the study level to avoid double counting. Study quality was appraised using the QA-SIVAS scale. **Results**: Non-contact mechanisms predominated in all studies reporting injury dynamics (64–100%). Acceleration from a stationary or near-stationary position was the most common movement context, with the “false step” frequently described as a recurrent initiating action rather than a singular cause. Ankle dorsiflexion at the injury frame was present across all studies (25.3–47.9°). A recurrent injury-frame configuration was observed, comprising trunk flexion, hip extension, knee extension or early flexion, ankle dorsiflexion, and a flat foot, reached through a progression from initial contact. **Conclusions**: ATR was predominantly a non-contact injury, most often during acceleration from a near-stationary position, reaching a broadly convergent injury-frame configuration. A rearward preparatory step recurred across studies and movement categories, but inconsistent definitions and incomplete reporting mean its overall frequency cannot be established. It remains a candidate movement feature for controlled biomechanical investigation, and future work should compare successful and injurious steps within athletes.

## 1. Introduction

The Achilles tendon (AT) is the strongest tendon in the human body, transmitting forces several times body weight between the triceps surae and the calcaneus [1]. Despite its substantial load-bearing capacity, the tendon remains susceptible to injury and catastrophic failure. A recent meta-analysis of 28 population-based studies involving more than 630 million individuals and approximately 563,000 Achilles tendon rupture (ATR) cases reported a nearly fivefold increase in global incidence, from 6.1 per 100,000 person-years in 1979 to 31.3 per 100,000 person-years in the early 2020s [2]. Approximately two-thirds of ATRs are associated with sporting activities, with the highest incidence occurring among men aged 30–49 years [2]. Basketball is identified as one of the sports most associated with ATR [3,4].

Achilles tendon rupture is one of the most consequential injuries in professional basketball. Between 72 and 78% of players return to play at all [5,6]. Those who do are typically out for roughly ten months [7,8], and a return to play rarely means a return to form. Only 27% regain their previous level of success [6], just over half remain in the league three years later [6], and careers shorten markedly (3.1 ± 2.3 vs. 5.8 ± 3.5 seasons [5]). The same decline appears in other professional sports after surgery [9]. Recent Achilles tendon ruptures involving high-profile National Basketball Association (NBA) players have therefore heightened interest in the injury’s circumstances and prevention. Beyond the individual athlete, ATR affects team performance and the commercial profile of professional basketball [10], and the number of documented confirmed ruptures in the league has increased. Confirmed ruptures rose to 22 in the 2010s from between four and ten per decade previously, with 14 already recorded in the first six seasons of the 2020s (Supplementary Table S1) [11]. These are unadjusted case counts and are not expressed relative to player exposure, so they describe documented cases rather than incidence.

Knowledge regarding ATR mechanisms was derived primarily from athlete reports, clinical observations, and epidemiological studies [2,12,13]. Although these approaches provided valuable information regarding injury frequency and broad activity classifications, they offered limited insight into the precise biomechanical events occurring immediately before rupture [14,15]. As a result, descriptions of injury mechanisms were often restricted to general categories such as running, jumping, landing, or changing direction [16,17].

The widespread availability of high-definition broadcast footage, together with advances in video-based motion analysis, has substantially improved the ability to investigate sports injuries. Video analysis permits frame-by-frame examination of movement kinematics during the injury event itself [16,17]. In the context of ATR, this approach enables researchers to move beyond broad activity descriptions and evaluate the kinematic characteristics associated with rupture occurrence.

Recent video-based investigations have begun to identify recurring biomechanical characteristics associated with ATR across multiple sports [7,18,19,20]. However, reported movement patterns and kinematic findings vary across studies because of differences in sporting context, analytical methodology, and measurement precision. Consequently, it remains unclear which biomechanical characteristics are consistently associated with rupture and which may be sport- or study-specific.

Despite increasing interest in video-based ATR research, the available literature remains fragmented. Studies have examined different sports, used varying analytical methods, and reported findings at different levels of kinematic precision, ranging from qualitative descriptions to quantitative joint angle measurements. Therefore, the purpose of this systematic review was to synthesize the available video analysis evidence describing the biomechanics of ATR in sport. Specifically, this review aimed to characterize the contact mechanism of rupture events, identify commonly reported movement patterns, summarize lower limb and whole body kinematics up to and at the injury frame (IF), and evaluate the consistency of biomechanical findings across different sporting contexts.

## 2. Materials and Methods

### 2.1. Study Design

This was a systematic review with a mixed-methods narrative synthesis, which was conducted in accordance with the PRISMA (Preferred Reporting Items for Systematic Reviews and Meta-Analyses) guidelines [21]. The review protocol was prospectively registered with PROSPERO (CRD420261425714). Given the anticipated heterogeneity in outcome measures, varying kinematic data precision, and the diverse nature of video-based observational data, a meta-analysis was deemed inappropriate. Narrative synthesis was therefore employed to provide a structured textual account of the findings, guided by the framework proposed by Popay et al. [22].

### 2.2. Search Strategy

A comprehensive literature search was conducted by two reviewers in the PubMed, Dimensions, OpenAlex, and Scopus electronic databases from database inception to June 2026. The searches were performed between 10 and 12 June 2026 and were designed to identify studies describing the biomechanics and injury mechanisms of acute ATR in sport using video-based analysis.

The following Boolean search strategy was initially developed for PubMed and subsequently adapted for the indexing and search functionality of the remaining databases:

(“Achilles tendon rupture” OR “Achilles tendon tear” OR “Achilles rupture” OR “AT rupture”) AND (sport OR athlete OR athletic OR competition OR training) AND (video OR footage OR “video analysis” OR “slow motion” OR film OR “image analysis” OR “image processing”) AND (biomechanics OR biomechanical OR kinematics OR kinematic OR mechanism OR “injury mechanism” OR “movement pattern”)

Where necessary, search syntax was adapted to accommodate the indexing and search functions of individual databases; in OpenAlex, the abbreviation “AT rupture” was removed, because the platform treats “AT” as a stop word and collapses the term to “rupture” (“Achilles rupture” retains coverage). The full search terms and record counts for each database are provided in Supplementary Table S2. Records identified from all databases were exported to Mendeley, where duplicate records were identified and removed.

To maximize search sensitivity and reduce the likelihood of missing relevant studies, the electronic database search was supplemented by a Google Scholar search (the first 100 of 2680 results, ranked by relevance, were screened) and by manual screening of the reference lists of all included studies and relevant review articles (snowballing).

No date restrictions were applied in order to capture the full historical breadth of video analysis research relating to ATR in sport. Searches were limited to studies published in English. The four databases searched combine a curated biomedical index (PubMed), a curated multidisciplinary index (Scopus), and two large open indexes with broad coverage that also capture conference material and records held outside subscription platforms (Dimensions and OpenAlex). Because the target literature is small and narrowly defined, this search was further supplemented by Google Scholar and by reference list screening of all included studies and relevant reviews. That screening returned no further eligible records, which suggests the search approached saturation for this topic.

### 2.3. Eligibility Criteria

The inclusion and exclusion criteria are presented in Supplementary Table S3. Studies were included if they documented acute, complete ATR confirmed through clinical or radiological assessment (Supplementary Table S3). Eligible cases required the injury to occur during verifiable athletic activity, with the inciting incident captured via video (e.g., broadcast, spectator, or surveillance footage). Furthermore, studies had to provide a detailed biomechanical or kinematic analysis of the movement patterns immediately preceding the rupture.

Studies were excluded if the injury mechanism was non-athletic, such as those resulting from accidental falls in daily life. Studies based solely on cadaveric models, laboratory simulations, or computational models without corresponding injury footage were excluded. Finally, to ensure data integrity, secondary sources such as reviews or meta-analyses were excluded unless they provided unique, original analysis of primary video data not reported elsewhere.

### 2.4. Study Selection

**Figure 1:**
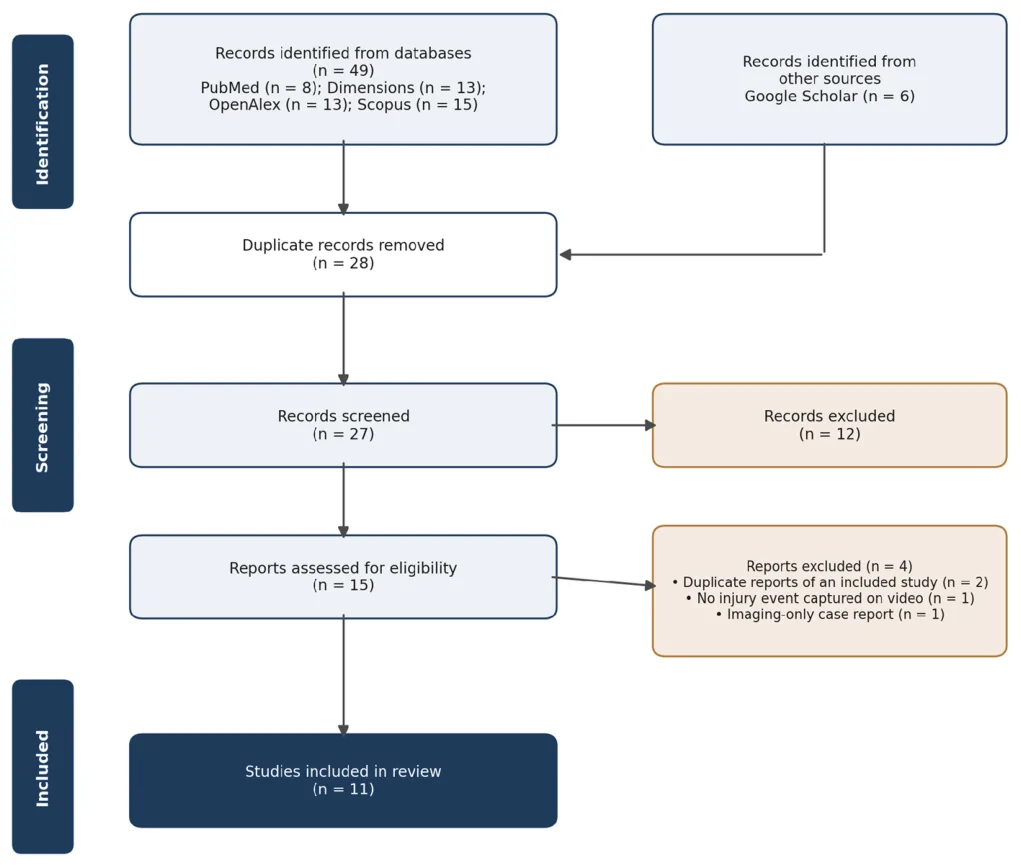
PRISMA 2020 flow diagram of study selection.

Following duplicate removal in Mendeley, all unique records were imported into Rayyan [23]. Two reviewers independently screened titles and abstracts against the predefined eligibility criteria, followed by full-text assessment of potentially eligible studies. Disagreements were resolved through discussion and consensus. The study selection process is summarized in Figure 1.

### 2.5. Data Extraction

Data extraction was performed by one reviewer using a standardized extraction form and independently verified by a second reviewer. Any discrepancies were resolved through discussion and consensus. Four biomechanical domains were targeted for extraction: contact mechanism (non-contact vs. contact), movement pattern in ATR (acceleration, change of direction, jumping, landing, and running/sprinting), joint and segment kinematics solely at the IF, and joint and segment kinematics from initial contact (IC) to the IF. The IF was defined as the specific video frame or moment in time identifying the onset of the ATR, operationalized by each study either as the initial peak in mechanical loading or as the visual identification of tendon failure. These two criteria are not equivalent and need not coincide. Peak dorsiflexion, tendon retraction, loss of balance, an athlete reaction, and an algorithmically detected kinematic change may each occur at different instants relative to actual tendon failure. The IF as reported should therefore be read as each study’s operational marker for rupture rather than as a verified instant of tissue failure. Data were extracted for the trunk, hip, knee, ankle, and foot in the sagittal plane, as well as the foot across all three planes. Recognizing that studies reported findings at varying levels of precision (quantitative, semi-quantitative, or qualitative), all available data were extracted and stratified accordingly to ensure a robust narrative synthesis.

### 2.6. Kinematic Data Classification

A priori, a three-tier classification framework was developed to account for the heterogeneity in kinematic reporting across studies. Quantitative data (Q) consisted of joint and segment angles reported as specific discrete values. Semi-quantitative data (SQ) were defined as kinematic data reported as ranges or thresholds rather than exact values. Finally, Qualitative (QL) data involved purely descriptive terms used to characterize joint positioning without numerical support. The classification was determined by the presence of exact angle values. Studies reporting solely the frequency of a joint or segment position (e.g., ankle dorsiflexion in 78% of cases) were classified as Qualitative, irrespective of the percentage value reported, as these figures describe the prevalence of a movement rather than the precision of the kinematic measurement itself. In addition to the precision tier, the anatomical reference used for each segment (a true joint angle, defined as the angle between the two adjoining segments, versus a segment to floor or segment to vertical reference) was recorded, as differing conventions affect the comparability of reported magnitudes across studies.

### 2.7. Injury-Frame Identification Taxonomy

**Figure 2:**
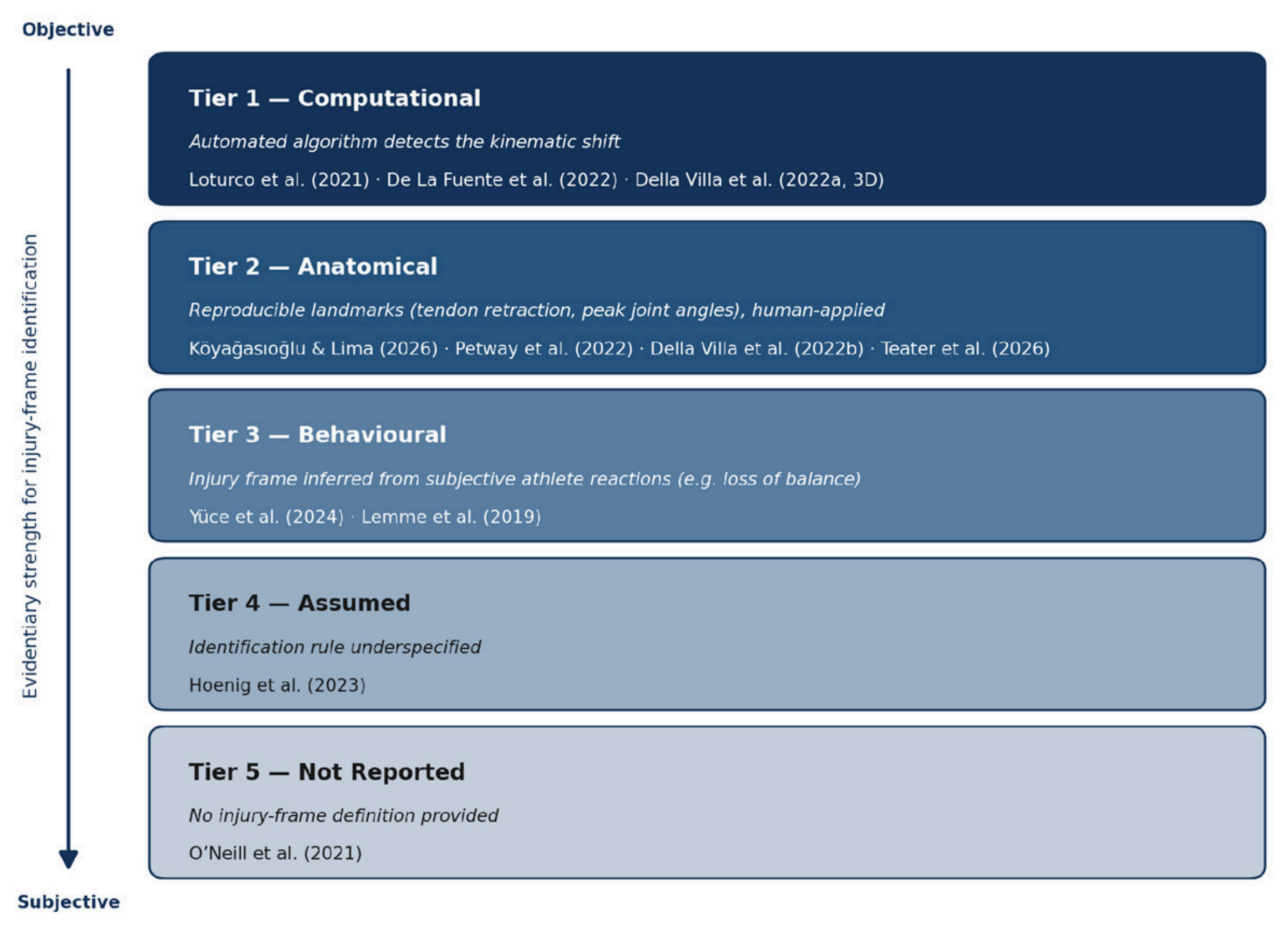
Proposed five-tier descriptive classification of injury frame (IF) identification methods, with study assignments. The tiers describe how the IF was identified and are not a validated ranking of evidentiary strength. Studies are shown in the figure by author and year; each study is cited by reference number in the accompanying text and tables.

To account for the varying methodological approaches observed across the literature, a novel five-tier taxonomy was developed to categorize the diverse strategies used for IF identification. The conceptual framework and study assignments for this taxonomy are visually detailed in Figure 2. The taxonomy describes how each study identified the IF. It is a proposed descriptive classification, not a validated ranking of evidentiary strength, and a higher tier does not mean a more valid identification. It reflects the reproducibility with which a frame could be selected, not the physiological plausibility that the selected frame is the true moment of failure, and not the temporal resolution or image quality of the footage. An algorithm may select a frame consistently yet still select the wrong event, whereas visible tendon recoil may lie closer to the tear but appear a few frames after it. Video quality is appraised separately as item 4 of the QA-SIVAS scale. The taxonomy is structured as follows. Tier 1 (Computational) utilized automated algorithms to detect kinematic shifts, while Tier 2 (Anatomical) relied on reproducible landmarks, such as tendon retraction or peak joint angles. Tier 3 (Behavioral) identified the IF through observer-identified athlete reactions, including loss of balance. Conversely, Tiers 4 (Assumed) and 5 (Not Reported) represented lower levels of methodological transparency, as identification rules were either underspecified or entirely absent.

### 2.8. Methodological Quality Assessment

Methodological quality was assessed using the Quality Appraisal for Sports Injury Video Analysis Studies (QA-SIVAS) scale [24], a validated tool developed specifically to appraise video-based sports injury studies. The scale comprises 18 items, each scored dichotomously as met (1) or not met (0), for a maximum of 18 points. Its items span the domains most relevant to the validity of video analysis research: the clarity of the study objective; the description of the sample and its characteristics; the source and quality of the analyzed video; the applied methods and use of a systematic analytical approach; medical confirmation of the diagnosis; the expertise and number of independent raters; the presence of a control or comparison condition; the validity and reliability of any quantitative measurements; the reporting of results, case numbers or proportions, and injury context; the provision of an example injury frame; and the adequacy of the discussion, clinical or practical implications, and stated limitations.

Each study’s total was expressed as a percentage of the maximum and categorized into one of four bands: low (<60%), moderate (60–70%), good (71–80%), or high (81–100%). Each study was independently appraised by two reviewers, with disagreements resolved through discussion and consensus, and inter-rater agreement for the appraisal was quantified using Cohen’s κ. Methodological quality judgements were not used as grounds for exclusion but to contextualize the interpretation of the biomechanical findings during narrative synthesis.

### 2.9. Data Synthesis

Synthesis was structured according to the predefined biomechanical domains and interpreted in relation to kinematic data classification, IF identification methodology, and methodological quality assessment (QA-SIVAS). The findings of the eleven included studies were synthesized narratively, following the Synthesis Without Meta-analysis (SWiM) reporting guideline [25], across the four layers of biomechanical analysis defined in Section 2.5 [22]. No meta-analysis was performed. Because several studies drew on overlapping player populations (within the NBA, professional soccer, and NFL cohorts), the included studies did not represent independent samples, and raw event counts could not be pooled without double-counting the same ruptures. The studies also varied too widely in their methodologies, outcome measures, and levels of kinematic detail to permit meaningful statistical pooling. Where studies reported joint angles as exact values in degrees, findings were compared directionally. Where studies described joint angles in general terms only, findings were compared descriptively.

## 3. Results

### 3.1. Study Selection and Characteristics

The database searches identified 49 records (PubMed, *n* = 8; Dimensions, *n* = 13; OpenAlex, *n* = 13; Scopus, *n* = 15), supplemented by 6 additional records identified through the Google Scholar search. Manual reference list screening (snowballing) identified no further eligible records. After removal of 28 duplicate records, 27 unique records were screened by title and abstract. Inter-rater agreement at this stage was high, with the few disagreements resolved through consensus discussion. Following title and abstract screening, 12 records were excluded, leaving 15 reports for full text eligibility assessment. Upon comprehensive full-text review, four reports were excluded based on the following specified criteria: duplicate reports of an already included study (*n* = 2), lack of an injury event captured on video footage (*n* = 1), and an imaging-only case report (*n* = 1).

A total of eleven studies met the inclusion criteria. Because several studies analyzed overlapping player populations (NBA, professional soccer, and NFL cohorts), the included studies did not represent independent samples. Consequently, injury events were synthesized at the study level rather than pooled across studies to avoid double counting the same ruptures. The eleven included publications do not correspond to eleven independent datasets. Supplementary Table S4 reports, for each study, the sport and competition, the period in which the analyzed ruptures occurred, the number of cases identified, the number with usable video, and the numbers entering qualitative and quantitative analysis, together with the other included studies whose samples are likely to overlap. Four publications report basketball cases from a single league across overlapping periods [7,20,26,27], and the window of Köyağasıoğlu and Lima [27] encompasses those of the other three. Two soccer publications [18,19] drew on the same injury database, applied the same restriction to the first and second national divisions and to in-competition injuries, and the period of the former falls entirely within that of the latter, so their samples are likely to overlap substantially. Yüce et al. [20] report basketball, American football and soccer cases between 1970 and 2020 and, therefore, intersect each of the other multi-case cohorts. Teater et al. [28] and the three single case reports [29,30,31] are the least likely to share events with the others. The eleven publications are therefore best understood as drawing on approximately four distinct athlete populations together with three individually reported cases, which is why events are synthesized at the study level and no pooled estimate is reported.

Study characteristics are summarized in Table 1. Three studies investigated NBA players, three examined professional soccer players, one analyzed NFL athletes, two included multisport cohorts, and two were single case reports in track and field [29,30]. Most studies involved male athletes; only two included female athletes, who represented a small minority of all reported cases.

### 3.2. Methodological Quality

Methodological quality was appraised with the Quality Appraisal for Sports Injury Video Analysis Studies (QA-SIVAS) instrument [24], comprising 18 binary items; the total (out of 18) was expressed as a percentage and categorized as low (<60%), moderate (60–70%), good (71–80%), or high (81–100%). Agreement between the two reviewers’ independent QA-SIVAS ratings was substantial (Cohen’s κ = 0.750; 95% CI 0.475 to 1.000). Per-study and item-level results are presented in Supplementary Table S5.

Overall methodological quality was moderate to good. Total scores ranged from 8 to 16 out of 18 (44.4–88.9%), with a mean of 12.9/18 (71.7%) and a median of 13/18 (72.2%). Two studies were rated high [19,30], five good [7,18,27,28,29], three moderate [20,26,31], and one low [32]. The single low rating reflects the conference abstract format of O’Neill et al. [32] rather than the conduct of the underlying analysis.

Reporting of the fundamentals was consistent: six items were satisfied by all eleven studies (a clearly stated objective, a comprehensive description of the applied methods, a systematic analytical approach, clearly described main results, the reporting of case numbers or proportions, and description of the injury context), and a further two by ten of eleven (discussion within the context of existing evidence; the articulation of clinical or practical implications). Quality differences therefore arose from a smaller set of discriminating items.

A control or comparison group (item 10) was present in only two studies, both single case reports comparing the injured with the contralateral limb [29,30]. Adequate documentation of the video source and quality (item 4) and medical confirmation of the diagnosis (item 7) were each met by three studies, reporting of rater background and expertise (item 8) by two, a quantitative analysis conducted with documented validity and reliability (item 11) by five, and an example injury-frame still (item 15) by seven. Every other study relied on publicly available or media sources without medical verification.

Two design-dependent items were unmet chiefly by the single case reports: a representative sample (item 2) and independent observation by two or more raters (item 9), each met by eight of eleven, reflecting study design rather than poor conduct.

### 3.3. Injury-Frame Identification

Injury frame (IF) identification approaches were classified within the five-tier taxonomy (Figure 2). Tier 1 (computational) included three studies [29,30,31], while Tier 2 included four studies [18,26,27,28]. Tier 3 comprised two studies [7,20], Tier 4 included one study [19], and Tier 5 included a single study [32], which did not report a definition of the IF.

### 3.4. Contact Mechanism

Across the two single athlete track and field cases [29,30], contact classification was not applicable due to the absence of opponent interaction. In the remaining nine studies, non-contact mechanisms predominated in all cohorts. Because several studies analyzed overlapping samples, event-level pooling was not performed to avoid double counting. Reported non-contact proportions ranged from 64% [28] to 100% [7], with variability primarily driven by differences in classification criteria within the Teater series (Table 2).

In the National Football League cohort, 49 of 77 injuries were classified as non-contact under a strict definition, with 27 further classified as indirect contact and one as direct contact [28]. Under a broader non-direct-contact definition, 76 of 77 cases were non-contact. The three-dimensional reconstruction case was also classified as non-contact [31].

### 3.5. Movement Patterns

Acceleration from a stationary or near-stationary position was the most frequently reported movement context across studies (Table 3), including 74% in Köyağasıoğlu and Lima [27], 67% in Lemme et al. [7], 42% in Della Villa et al. [18], 40.5% in Yüce et al. [20], and 36.3% in Hoenig et al. [19], the latter combining their stepping back and starting categories. In Yüce et al. [20], the margin was narrow, with a stop and turn category accounting for a further 38.1%. The three single case reports fell outside this pattern, comprising the absorption phase of a bilateral drop jump [29], a high jump run-up [30], and a crossover cut [31], while O’Neill et al. [32] did not report sufficient classification detail. In the National Football League cohort, change of direction was most common (40%), with acceleration initiated by a rock-back accounting for 27% of cases [28]. Change of direction was not confined to that cohort. It was almost as frequent as acceleration in the multisport cohort of Yüce et al. [20] and was also reported in basketball and soccer [7,18,31].

Figures are as reported by each study and dashes indicate the action was not separately reported. Task categories follow the strata proposed during peer review. Because each study applied its own movement classification, events were mapped onto these categories by the review authors rather than reported in this form by the original studies, and frequencies are not additive across categories. Contact conditions are reported in Table 2, because contact cross-cuts movement category rather than forming one, and the indirect contact events of Teater et al. [28], described there as an overload scenario accounting for 30% of that cohort, appear in that table. Lemme et al. [7] reported pivoting rather than a named change of direction task, and its discussion states 8%, whereas its results table reports 2 of 12. Yüce et al. [20] are reported here from their results table, which gives stop and rotate as 16 of 42 (38.1%); their discussion and abstract state 38.5%. The other category of Della Villa et al. [18] contains four landing, four sidestep and one stretching case; the sidestep cases are a change of direction technique but are retained here within that category rather than reclassified. Köyağasıoğlu and Lima [27] additionally classified events on a basketball-specific action axis of cross-overs, catching a pass and pushing in, which cannot be combined with the categories used here. Petway et al. [26] reported no task classification, describing all analyzed cases as a backward step with the injured limb posterior to the center of mass. Hoenig et al. [19] describe stepping back, starting and jumping as related and overlapping patterns, which together account for 48.8% of that cohort. They separately recorded direction of movement, which was backward in 34 of 80 injuries (42.5%). That variable records the direction in which the player was travelling rather than the placement of the limb, so it captures their stepping back and jumping injuries together with three landing injuries, but not their starting injuries, in which players were accelerating forward.

The configuration at the injury frame was consistent across categories, comprising trunk flexion, hip extension, knee extension or early flexion, and ankle dorsiflexion with a flat or pronated foot, reached in a closed chain with the injured limb loaded. What differed between categories was the action leading into that configuration. Across studies reporting finer movement structure, acceleration from a stationary position was typically initiated by a backward preparatory step (“false step”), defined as a rearward placement of the foot posterior to the center of mass prior to forward propulsion [19,26]. This action was observed in 27–100% of cases where explicitly assessed across basketball, soccer, and American football cohorts [18,19,26,27,28]. These figures are not independent observations. The two basketball cohorts [26,27] draw on overlapping National Basketball Association populations, as do the two soccer cohorts [18,19], so the range reflects five publications rather than five independent samples.

The backward step was not restricted to a single movement category but appeared across multiple classifications within studies. For example, Hoenig et al. [19] reported stepping back as the most common pattern (26%) and described it as related to and overlapping with their starting and jumping patterns, which together account for 48.8% of that cohort; Della Villa et al. [18] identified it as a defining feature of acceleration from standing and jumping duels; and Teater et al. [28] described a rock-back pattern in 27% of cases, while noting a backward placement of the injured limb in most injuries regardless of category. Accordingly, reported frequencies likely represent a lower bound of its occurrence rather than its full prevalence. Terminology also varied across studies. False step, stepping back, rock back, and backward placement of the injured limb were used to describe related but not necessarily identical actions, which limits direct comparison of the reported frequencies.

### 3.6. Kinematics up to and at the Injury Frame

Across the eleven included studies, kinematic data were reported at three levels of precision (quantitative, semi-quantitative, and qualitative). A recurrent injury-frame configuration was observed in the majority of studies, characterized by trunk forward flexion, hip extension, knee extension or early flexion, ankle dorsiflexion, and a flat foot position in the sagittal plane (Table 4).

At the IF (Table 4), trunk flexion was reported in seven studies. Among the vertically referenced quantitative cohorts, it ranged from approximately 15° to 28° [18,27,31], while Petway et al. [26] reported a larger value (≈54°) measured relative to the hip axis, which is not directly comparable. Semi-quantitative and qualitative reports indicated trunk flexed in 50–73.8% of cases [19,20] and anterior orientation relative to the center of mass [28].

Hip kinematics were reported in seven studies, all showing hip extension, although the magnitudes are not directly comparable owing to differing reference frames [18,27]; Della Villa et al. [31] also described hip extension without reporting a sagittal angle. Semi-quantitative and qualitative findings indicated hip extension in 61.9–86% of cases [7,19,20,28,32].

Knee kinematics were reported in ten studies. Across the acceleration from standstill cohorts, injury-frame flexion clustered at approximately 22–27° [18,27,31], while a single high jump running case reported greater flexion of 41° at rupture [30]. Semi-quantitative classifications indicated <45° flexion in 97–100% of cases [7,19], and qualitative reports described knee extension or early flexion across cohorts [28,29,32]. Extension and early flexion are treated together because below approximately 45 degrees of knee flexion the gastrocnemius remains lengthened across both joints when the ankle is dorsiflexed, whereas a deeply flexed knee slackens it.

Ankle kinematics were reported in all eleven studies, with dorsiflexion at the IF ranging from approximately 25° to 48° across quantitative studies [18,26,27,30,31], while semi-quantitative and qualitative reports indicated dorsiflexion in 71–100% of cases across cohorts [7,19,20,28,29,32].

Foot kinematics were less reported. A flat foot position in the sagittal plane was observed in 62–85% of cases where assessed [18,27]. In the transverse plane, external rotation or abduction predominated in 69–97% of cases across three studies [18,19,26], whereas neutral positioning was most common in one cohort [7]. In the coronal plane, pronation predominated in 48–98% of cases in some studies [18,19], while neutral positioning was reported in 75–76% of cases in others [7,20]. The only three-dimensional reconstruction study additionally quantified approximately 10° of pronation and 15° of external rotation [31].

Reference conventions for reporting joint angles differed across the quantitative studies, which determined whether the reported magnitudes could be compared directly (Table 5). Trunk angle was vertically referenced (upright = 0°, flexion positive) in Della Villa et al. [18] and Köyağasıoğlu and Lima [27]. Petway et al. [26] measured the trunk relative to the hip axis, accounting for its larger value and precluding direct comparison. The hip was measured as a true intersegmental joint angle (0° = trunk and thigh aligned, flexion positive) by Della Villa et al. [18] and as a binned range by Hoenig et al. [19], whereas Köyağasıoğlu and Lima [27] measured the thigh relative to the floor. Magnitudes are therefore not directly comparable, and the finding is reported by direction. The knee was measured as a true joint angle (0° = thigh and shank aligned, flexion positive) across the quantitative studies, so these values are comparable. The ankle was measured with 0° defined as the foot perpendicular to the shank (dorsiflexion positive), and the values are directly comparable, ranging from approximately 25° to 48° (25.3–47.9°).

Quantitative analyses demonstrated progressive changes from initial contact to IF in trunk (≈10–18° to 15.5–27.5°), knee (≈42–44° to 22–27°), and ankle (≈0–6° plantarflexion to 29–42° dorsiflexion) [18,27,31]. The hip extended across the quantitative cohorts, but the magnitudes are not directly comparable owing to differing reference frames [18,27,31] (Table 6). The same directions were reported at lower precision. Hoenig et al. [19] recorded movement from flexion to extension at the knee in 92% of cases and from plantarflexion to dorsiflexion at the ankle in 78%, while Teater et al. [28] and Petway et al. [26] described the same transitions qualitatively. The knee therefore moved toward extension in every study that reported the transition.

## 4. Discussion

Across the eleven studies, the contact mechanism and the kinematics converged, the movement contexts converged less completely, and the interpretations diverged. Authors agreed that rupture was predominantly non-contact and that it occurred in a broadly convergent configuration at the injury frame, reached through a consistent progression from initial contact. Acceleration or takeoff from a near-stationary position was the most frequently reported movement context in almost every cohort, although change of direction predominated in the National Football League cohort, and authors diverged in their biomechanical explanations of how and why those kinematics led to tendon failure. To the authors’ knowledge, this systematic review represents one of the first mixed-methods syntheses of video-based ATR evidence across multiple sports, precision levels, and analytical frameworks.

The finding that ATRs are overwhelmingly non-contact, ranging from 64% to 100% across studies, is mechanistically expected. The force that ruptures the tendon is the high internal tension the athlete’s own triceps surae generates and transmits through the Achilles as it contracts against the planted foot, and rupture follows when that internally produced force overcomes the tendon’s mechanical limit [18]. No collision from an opponent is required, so the predominance of non-contact injuries is the expected consequence.

If the rupturing force is generated internally, the question becomes which movement generates enough of it to reach the tendon’s limit. Across the review, that context was consistent.

The most frequently reported movement context was explosive acceleration or takeoff from a near-stationary position, although change of direction was the largest category in the National Football League cohort [28] and almost as frequent as acceleration in the multisport cohort [20]. A rearward false step recurred as a preparatory action across different movement types. Across basketball, soccer, and American football, it was observed before accelerations, jumping actions, and changes of direction, suggesting that its contribution is greater than the discrete category frequencies of 26 to 27% reported by Hoenig et al. [19] and Teater et al. [28]. Hoenig et al. [19] support this directly. Their discrete stepping back category accounts for 26% of injuries, but they describe stepping back, starting and jumping as related and overlapping patterns, which together account for 48.8%, and they separately record backward-directed movement in 42.5% of the cohort. Della Villa et al. [18] likewise describe the action as a defining feature of two separate patterns. The spread of these observations across different sports is the stronger part of this argument, because the basketball reports draw on overlapping league populations and therefore do not provide independent replication within that sport.

Because the rupturing force is internal, the question is what makes it large enough to exceed the tendon’s limit, and two explanations have been proposed. The first is acute mechanical overload, arising from three contributing features. During the false step, the athlete plants the injured limb behind the center of mass, and as the triceps surae contracts eccentrically against that now planted foot, the force transmitted through the Achilles approaches six to eight times body weight, which can exceed its ultimate strength [7,20]. That force is amplified because the forward trunk and hip carry the center of mass anterior to the foot, lengthening the moment arm about the ankle that the plantarflexors must resist [27,28]. It is amplified further by the configuration reached at the injury frame itself, in which the knee is extended and the ankle dorsiflexed, considered below. On this account, the danger of the false step lies less in its velocity than in its configuration, since a high internal tendon force carried on one limb across a long lever can exceed the tendon’s limit even when the movement looks unremarkable [26,28]. The second explanation shifts the emphasis to the tendon itself, proposing that rupture reflects reduced tissue capacity rather than an excessive external moment, since these actions occur at low velocities that would not usually overload a healthy tendon, which implicates preexisting tendinopathy and tendon degeneration [18]. These accounts are mechanistic inferences rather than measurements. The included studies provide segment kinematics estimated from broadcast footage, not tendon force, ankle joint moments, gastrocnemius fascicle behavior, muscle activation, tendon strain, or the direction of muscle-tendon work. The loading magnitudes cited above are drawn from the wider biomechanics literature rather than from the injury events themselves, so the mechanisms proposed here should be read as hypotheses that video analysis alone cannot test.

These accounts describe the whole-body demand. To see how that demand acts on the tendon, it helps to follow the joint and segment motion from initial contact to the injury frame, and then the posture reached at the frame itself.

Studies capturing the transition from initial contact to the injury frame describe a similar movement, with the ankle moving from plantarflexion or neutral into dorsiflexion, the knee and hip from flexion toward extension, and the trunk into deepening forward flexion. As the ankle is driven into dorsiflexion while the triceps surae contracts to keep the body from toppling over the planted foot, the plantarflexors lengthen under tension in a way consistent with eccentric loading [7,18,20,32], storing elastic energy within the tendon in a stretch-shortening cycle [33]. O’Neill et al. [32] captured this convergence directly, reporting high-load eccentric plantarflexor activity, ankle dorsiflexion, and concurrent knee extension in every case and hip extension in 86%. The dominant loading mode is not settled, however. Hoenig et al. [19] proposed that overload may instead arise from the interaction between proximally generated concentric triceps surae force and externally imposed ankle dorsiflexion rather than eccentric lengthening alone. This does not overturn the consensus that the tendon is heavily loaded through the transition, but it leaves the precise mechanical mode at the instant of rupture unresolved.

Regardless of which loading mode predominates, the transition from initial contact to the injury frame delivers the limb into a consistent end posture, and the length-tension consequences of that posture offer one explanation for the high tendon loading. With the knee extended and the ankle dorsiflexed, the biarticular gastrocnemius is stretched across both joints at once, placing it near the peak of its length-tension relationship and maximizing the tension it develops [34,35]. It is in this lengthened, high-tension state that the explosive propulsive demand of takeoff is delivered to the muscle-tendon unit [36]. The ankle sits at end-range dorsiflexion, directly elongating the muscle-tendon unit to a degree that may approach or exceed the physiological weight-bearing range [18,26,29], a strain compounded by reduced knee flexion and impaired knee-to-ankle coordination [30].

One caveat qualifies this otherwise consistent picture. The injury posture is derived almost entirely from sagittal plane analyses, as ten of the eleven studies relied on two-dimensional video. The only direct evidence of multiplanar loading [31] identified substantial frontal and transverse plane motion, with the trunk rotated laterally toward the injured side, the hip adducted, and the ankle externally rotated and pronated over a foot planted medial to the center of gravity. However, this case involved a crossover cut rather than a false step, suggesting that out-of-plane loading may be scenario-dependent and more pronounced during change-of-direction injuries. Whether these multiplanar motions are incidental or contribute independently to tendon failure cannot be determined from a single case, which highlights an important methodological gap in the current evidence base.

This review addressed how ruptures occur rather than why their frequency appears to be rising. Intrinsic and contextual factors fall outside the scope of a video analysis synthesis but remain important for both athletic and general populations. Reported risk factors for Achilles tendon rupture include age, sex, prior tendinopathy, systemic disease, footwear, genetics and medication use [19], and the incidence of Achilles tendon rupture has risen over several decades [2,12]. Video analysis cannot establish whether changes in footwear, denser competitive schedules, or the management of minor tendon complaints in the period before rupture contribute to that trend, because none of these are visible in broadcast footage and none were reported by the included studies. Linking video-derived movement patterns to tendon health, training load and treatment history is therefore a priority for future work.

There were limitations in this review. Methodological heterogeneity precluded meta-analysis. Eligibility was restricted to English language studies, introducing potential language bias, and several studies analyzed overlapping samples; therefore, contact mechanism is presented as a study-level range rather than a pooled proportion, with Wilson score confidence intervals reported for individual studies. Most studies relied on publicly available two-dimensional broadcast footage, with variable reporting of video quality and limited medical confirmation of diagnosis; only one study performed three-dimensional reconstruction [31]. Nine of eleven studies included male athletes only, and one conference abstract [32] was retained despite its methodological limitations because of its relevance. All data attributed to that report were extracted from the published abstract itself. The authors were contacted for additional methodological detail but did not respond, so no unpublished data were obtained, and the record was appraised exactly as published, which is reflected in its quality rating. Three databases commonly used in sports medicine and biomechanics, namely Web of Science, SPORTDiscus and Embase, were not searched, and relevant records indexed only on those platforms may therefore have been missed. The Google Scholar component should also be regarded as a pragmatic supplement rather than a reproducible exhaustive search, because relevance ranking varies with date, location and search history, so the first 100 results do not constitute a stable retrievable set.

### Future Directions

Several important questions remain unresolved. First, research is necessary to identify if ATRs are driven by an unusually demanding movement execution, reduced tendon capacity, or an interaction between the two. Video analysis alone cannot distinguish between these competing explanations. Second, future research should compare rupturing movements with non-injurious executions of the same task to identify the biomechanical characteristics that distinguish injury from successful performance. Within-athlete comparisons may be particularly valuable because they minimize between-subject differences in tendon morphology, training history, and other intrinsic factors while isolating differences in movement execution. Third, while the backward (“false”) step was identified as a recurring preparatory action, no study directly quantified how variations in false step execution influence AT loading or subsequent injury risk. Prospective studies are therefore needed to determine whether specific biomechanical characteristics are associated with increased tendon loading and whether these factors represent modifiable targets for injury prevention. Finally, the precise loading mechanism at the instant of rupture remains uncertain. Integrating video analysis with musculoskeletal modeling, wearable technologies, or subject-specific biomechanical simulations may help improve understanding of the mechanical events leading to tendon failure.

## 5. Conclusions

The available evidence was limited in quantity and consisted predominantly of observational video analyses, restricting causal inference. Nevertheless, findings converged in identifying ATR as a predominantly non-contact injury, most commonly occurring during acceleration from a near-stationary position, although change of direction was the largest category in the National Football League cohort. The configuration reached at the injury frame was broadly convergent, comprising trunk flexion, hip extension, knee extension or early flexion, and ankle dorsiflexion with a flat or pronated foot. A rearward preparatory step recurred across cohorts and movement categories and represents a candidate movement feature for controlled biomechanical investigation, although the included studies defined it inconsistently and reported it incompletely, so its frequency across all ATR events cannot be established. The relative contributions of acute mechanical overload and preexisting tendon degeneration also remain unresolved. Future research should combine biomechanical analysis of rupturing and non-injurious movements with assessment of tendon health to better distinguish modifiable movement patterns from intrinsic tissue susceptibility and inform injury prevention.

## Figures and Tables

**Table 1:** Characteristics and QA-SIVAS methodological quality appraisal of the eleven included studies.

					QA-SIVAS Methodological Quality		
Study	Sport/League	Design	ATR Events (*n*)	Sex	Total/18	%	Band
Hoenig et al. [19]	Soccer	Case series	80	Male	16	88.9%	High
De La Fuente et al. [30]	Track and field (high jump)	Single case report	1	Male	15	83.3%	High
Teater et al. [28]	American football (NFL)	Case series	77	Male	14	77.8%	Good
Della Villa et al. [18]	Soccer	Case series	60	Male	14	77.8%	Good
Loturco et al. [29]	Track and field (sprint)	Single case report	1	Female	14	77.8%	Good
Lemme et al. [7]	Basketball (NBA)	Case series	12	Male	13	72.2%	Good
Köyağasıoğlu & Lima [27]	Basketball (NBA)	Case series	27	Male	13	72.2%	Good
Petway et al. [26]	Basketball (NBA)	Case series	13	Male	12	66.7%	Moderate
Yüce et al. [20]	Multisport	Case series	42	Mixed	12	66.7%	Moderate
Della Villa et al. [31]	Soccer (UEFA Euro)	Single case report (3D)	1	Male	11	61.1%	Moderate
O’Neill et al. [32]	Multisport	Case series (conference abstract)	57	Male	8	44.4%	Low
**Total**	**11 studies**	**—**	**—**	**>99% male**	**12.9**	**71.7%**	**—**

Median 13/18 (72.2%); range 8–16 (44.4–88.9%). Bands: low < 60%, moderate 60–70%, good 71–80%, high 81–100%. Bold indicates the summary row, which reports totals and means across the eleven studies rather than data from an individual study. Em dashes in the summary row indicate cells for which a total or mean is not applicable. Note. Sport/league and design as reported by each study. Della Villa et al. [31] is the single three-dimensional reconstruction; the remaining studies used two-dimensional video. O’Neill et al. [32] is a multisport conference abstract (field and other sports; *n* = 57). Event counts are reported per study; because several studies analyzed overlapping player populations, these counts cannot be summed to a unique total without double-counting individual ruptures.

**Table 2:** Contact mechanism across the nine included studies.

Study	Non-Contact (95% CI)	Contact/Indirect Contact	*n*	Sport
Lemme et al. [7]	100% [75.8–100]	0%	12	Basketball
Köyağasıoğlu & Lima [27]	81.5% [63.3–91.8]	18.5%	27	Basketball
Petway et al. [26]	92% [66.7–98.6]	8%	13	Basketball
Della Villa et al. [18]	83% [72.0–90.7]	17% indirect	60	Soccer
Della Villa et al. [31]	100% [20.7–100]	0%	1	Soccer
Hoenig et al. [19]	80% [70.0–87.3]	20% (direct + indirect)	80	Soccer
Yüce et al. [20]	83.3% [69.4–91.7]	16.7%	42	Multisport
O’Neill et al. [32]	91% [81.1–96.2]	9%	57	Multisport
Teater et al. [28]	64% [52.5–73.5]	36%	77	American football (NFL)

The two single-athlete track and field cases [29,30] are excluded, as a contact mechanism is not applicable. Values are strict non-contact proportions calculated within each study. Intervals are Wilson score 95% confidence intervals for a binomial proportion, chosen in preference to the normal approximation because several studies report small denominators or proportions at or near 100%, where the normal approximation performs poorly and can return impossible bounds. Each interval describes the precision of that study’s own estimate only. No pooled estimate is reported because the cohorts analyze overlapping samples. For Della Villa et al. [31], where *n* = 1, the interval spans almost the entire possible range and should be read as an indication of the absence of precision rather than as a meaningful estimate. Non-contact mechanisms predominated in every study, ranging from 64% [28] to 100% [7,31].

**Table 3:** Movement patterns by task category across the included studies.

Study	Stationary or Near Stationary Acceleration	Change of Direction	Running or Sprinting	Jump Takeoff or Landing	Backward Preparatory Step
Köyağasıoğlu & Lima [27]	74%	—	—	22.2% (jump 14.8%, landing 7.4%)	Most cases, within take-off/acceleration
Lemme et al. [7]	67%	17% (pivoting)	—	17% (jumping 8%, landing 8%)	—
Della Villa et al. [18]	42%	25% (cross-over cut)	—	25% (jumping 18%, landing 7%)	60% (acceleration and jumping patterns, where a backward step is described as typical)
Yüce et al. [20]	40.5%	38.1% (stop and turn)	—	21.5% (landing 16.7%, jump 4.8%)	—
Hoenig et al. [19]	36.3% (stepping back 26%, starting 10%)	—	18%	33% (jumping 13%, landing 20%)	26% stepping back; 48.8% including starting and jumping, which the authors describe as related patterns
Teater et al. [28]	27% (rock back)	40%	—	1% (landing)	Most cases, regardless of movement category
Petway et al. [26]	All 13 analyzed cases; no task classification reported	—	—	—	100% (13 of 13)
Della Villa et al. [31]	—	Single case (cross-over cut)	—	—	—
Loturco et al. [29]	—	—	—	Single case (drop jump absorption)	—
De La Fuente et al. [30]	—	—	Single case (high jump run-up)	—	—
O’Neill et al. [32]	Insufficient detail to classify	—	—	—	—

**Table 4:** Joint and segment configuration at the injury frame.

Study	Trunk	Hip	Knee	Ankle	Foot (All Planes)
Petway et al. [26]	54.2° †	NR	NR	47.9° DF	69% ext rot
Köyağasıoğlu & Lima [27]	27.5°	26.8° ext ‡	26.7° flex	41.6° DF	85% flat (sagittal)
Lemme et al. [7]	NR	83% ext	0–45° flex (100%)	75% DF	Neutral (axial 67%, coronal 75%)
Della Villa et al. [18]	15.5°	−19.5° (19.5° ext)	22.5° flex	40° DF	62% flat; 48% pronation; 70% ext rot
Della Villa et al. [31]	23° flex	NR	24° flex	29° DF	Pronation
Hoenig et al. [19]	Forward 50%	<45° in 92%	<45° in 97%	78% DF	Pronation 98%; Abduction 97%
Teater et al. [28]	Anterior lean (qual.)	Extension (qual.)	Extension (qual.)	Peak DF (qual.)	NR
Yüce et al. [20]	73.8% flex	61.9% ext	54.8% ext	71.4% DF	Neutral (coronal 76.2%)
O’Neill et al. [32]	NR	86% ext	100% ext	100% DF	NR
De La Fuente et al. [30]	NR	NR	25.3° flex	41.1° DF	NR
Loturco et al. [29]	NR	NR	Hyperextension (qual.)	Elevated DF (qual.)	Eversion (qual.)

Quantitative values in degrees; percentages denote categorical or binned frequencies; NR = not reported; qual. = qualitative. DF = dorsiflexion; ext = extension; flex = flexion. † Petway measured the trunk relative to the hip axis, not vertical, so its value is not comparable. ‡ Köyağasıoğlu & Lima measured the thigh relative to the floor.

**Table 5:** Joint angle measurement conventions across the eleven studies.

Study	Tier	Ankle	Knee	Hip	Trunk
Köyağasıoğlu & Lima [27]	Q	True joint angle (shank–sole); DF+	True joint angle (thigh–shank); flex+	Thigh-to-FLOOR (segment-ref.)	Vertical-referenced; flex+
Della Villa et al. [18]	Q	True joint angle; DF+	True joint angle; flex+	True intersegmental joint angle	Vertical-referenced; flex+
Della Villa et al. [31]	Q (3D, *n* = 1)	True joint angle (3D); DF+	True joint angle (3D); flex+	True 3D joint angle; ext/add	3D segment angle; flex + rotation
Petway et al. [26]	Q	DF angle, injured leg	Categorical; 67.4° = NON-injured lead leg	Categorical (HE/HF), injured leg	Relative to hip axis—not comparable
De La Fuente et al. [30]	Q (*n* = 1)	True joint angle (cosine theorem); DF+	True joint angle; flex+	Not reported	Not reported
Hoenig et al. [19]	SQ	Categorical (PF/neutral/DF)	Joint angle BIN (<45/45–90/>90°)	Joint angle BIN; ext = <45° flex	Categorical, earth-vertical ref.
Teater et al. [28]	QL	Categorical (peak DF)	Qualitative (ext)	Qualitative (ext)	Qualitative (anterior lean)
Lemme et al. [7]	QL	Categorical sagittal	Categorical bin ‘0–45°’	Categorical (ext/flex)	Not reported
Yüce et al. [20]	QL	Categorical	Categorical (ext/flex)	Categorical	Categorical (forward)
O’Neill et al. [32]	QL	Categorical	Categorical	Categorical	Not reported
Loturco et al. [29]	Image (*n* = 1)	DF–PF trace; no anatomical zero	Qualitative note	Not reported	Not reported

Tier: Q = quantitative; SQ = semi-quantitative (binned); QL = qualitative. A true joint angle is measured between the two segments forming the joint; segment-referenced angles are measured against the floor or vertical. Ankle and knee are comparable; hip is reported by direction; trunk is comparable only among the vertical-referenced studies. DF = dorsiflexion; PF = plantarflexion; 3D = three-dimensional.

**Table 6:** Joint and segment motion from initial contact (IC) to injury frame (IF).

Study	Trunk (IC → IF)	Hip (IC → IF)	Knee (IC → IF)	Ankle (IC → IF)
Köyağasıoğlu & Lima [27]	18.2° → 27.5°	12.3° → 26.8°	42.2° → 26.7°	5.6° PF → 41.6° DF
Della Villa et al. [18]	15° → 15.5°	10° → −19.5°	44° → 22.5°	0° → 40° DF
Della Villa et al. [31]	10° → 23° (+rotation)	→ ext	→ 24° flex	→ 29° DF
Hoenig et al. [19]	Forward (held)	Flexion → extension (83%)	Flexion → extension (92%)	PF → DF (78%)
Teater et al. [28]	Anterior lean (qual.)	→ extension (qual.)	→ extension (qual.)	→ peak DF (qual.)
Petway et al. [26]	Lowering of trunk (qual.)	NR	NR	PF → end range DF

Paired IC → IF values (quantitative) or direction of movement with frequency (Hoenig)/qualitative description (Petway). Across reporters, the ankle moved from plantarflexion or neutral into dorsiflexion while the knee and hip moved from flexion toward extension, with the trunk held in forward flexion. Hip values for Köyağasıoğlu and Lima [27] are thigh-to-floor angles rather than true joint angles, so the increase from 12.3° to 26.8° represents increasing extension. IC = initial contact; IF = injury frame; DF = dorsiflexion; PF = plantarflexion.

## Data Availability

No new data were created or analyzed in this study. All data supporting the findings of this review are contained within the cited published studies.

## Supplementary Materials

The following supporting information can be downloaded at: https://www.mdpi.com/article/10.3390/jfmk11030309/s1, Table S1: Confirmed NBA Achilles tendon ruptures by decade (1970–2025); Table S2: Database search strategy; Table S3: Inclusion and exclusion criteria; Table S4: Unit of analysis and cohort overlap across the eleven included studies; Table S5: QA-SIVAS methodological quality appraisal of the eleven included studies.
