# Supplementary material for: Acceleration and Injury-Frame Biomechanics in Achilles Tendon Rupture: A Systematic Review
Source: J Funct Morphol Kinesiol. 2026 Aug 8;11(3):309. doi: 10.3390/jfmk11030309 (PMC13510858; doi:10.3390/jfmk11030309)
Supplement: Supplementary file 1 [file jfmk-11-00309-s001.zip › jfmk-4463233-supplementary.pdf]

Supplementary Material

Table S1. Confirmed NBA Achilles tendon ruptures by decade (1970–2025).

Decade	n	Confirmed players
1970s	5	Bob Rule; Elgin Baylor; Eddie Miles; Phil Smith; Nate ‘Tiny’ Archibald
1980s	4	Kevin Porter; Calvin Natt; Gene Banks; Norm Nixon
1990s	10	Dominique Wilkins; Isiah Thomas; Gerald Wilkins; Stanley Roberts; Felton Spencer; Scott Burrell; David Benoit; Laphonso Ellis; Derrick McKey; Christian Laettner
2000s	8	Maurice Taylor; Don Reid; Emanuel Davis; Courtney Alexander; Voshon Lenard; Dan Dickau; Laron Profit; Elton Brand
2010s	22	Mehmet Okur; DeSagana Diop; Darrell Arthur; Chauncey Billups; Elliott Williams; Jerome James; Kobe Bryant; Jeffrey Taylor; Anderson Varejão; Xavier Henry; Brandon Jennings; Wesley Matthews; Mario Chalmers; Rudy Gay; Sheldon Mac; DeMarcus Cousins; C.J. Wilcox; J.J. Barea; John Wall; Kevin Durant; Rodney Hood; David Nwaba
2020s	14*	Dwight Powell; Klay Thompson; Chris Clemons; Edmond Sumner; Brandon Clarke; DaRon Holmes II; Isaiah Jackson; James Wiseman; Dru Smith; Thanasis Antetokounmpo; Dejounte Murray; Damian Lillard; Jayson Tatum; Tyrese Haliburton
**Total**	**63**	Confirmed complete NBA Achilles tendon ruptures, 1970–2025

Strictly limited to confirmed complete ruptures/tears; partial tears and tendinopathy excluded. *2020s reflects the first six seasons (14 confirmed) and is not projected to a full decade total. Sources: Pro Sports Transactions; NBA.com; ESPN; The Athletic; newspaper archives; NBA Advanced Stats [11].

Table S2. Database search strategy.

Source	Search terms	Field	Records
PubMed	Achilles tendon rupture AND (video OR biomechanics OR kinematics OR mechanism)	Title/Abstract	8
Dimensions	As above, adapted to database syntax	Full text	13
OpenAlex	As above, adapted to database syntax	All fields	13
Scopus	As above, adapted to database syntax	Title/Abstract/Keywords	15
Google Scholar	Achilles tendon rupture video analysis biomechanics sport	All fields (first 100 of 2,680 screened)	6
Reference list screening (snowballing)	Reference lists of included studies and relevant reviews	Manual	0 (no new records)
**Total identified**	55 identified → 28 duplicates removed → 27 screened → 15 full text → 11 included		**55**

Four databases (PubMed, Dimensions, OpenAlex, Scopus) searched from inception to June 2026 (searches run 10–12 June 2026), supplemented by a Google Scholar search (first 100 of 2,680 results screened by relevance) and reference list screening (snowballing). English language only; no date restriction. Reporting follows the PRISMA 2020 statement [21].

Table S3. Inclusion and exclusion criteria.

Inclusion criteria	Exclusion criteria
• Acute, complete Achilles tendon rupture confirmed by clinical or radiological assessment<br>• Injury occurred during verifiable athletic activity<br>• Inciting incident captured on video (broadcast, spectator, or surveillance footage)<br>• Detailed biomechanical or kinematic analysis of the movement patterns immediately preceding the rupture	• Non-athletic injury mechanism (e.g. accidental falls in daily life)<br>• Studies based solely on cadaveric models, laboratory simulations, or computational models without corresponding injury footage<br>• Secondary sources (e.g. reviews or meta-analyses) unless providing unique, original analysis of primary video data not reported elsewhere

Applied at title/abstract and full text screening stages.

Table S4. Unit of analysis and cohort overlap across the eleven included studies.

Study	Sport and competition	Period of injuries	ATR cases identified	Cases with usable video	n qualitative	n quantitative	Included studies whose samples are likely to overlap
Lemme et al. [7]	Basketball (NBA)	1969–70 to 2017–18 seasons	44	12	12	0	[20], [26], [27]
Della Villa et al. [18]	Soccer, first and second national divisions	August 2008 to August 2018	118 (86 in official matches)	60	60	42	[19], [20]
Hoenig et al. [19]	Soccer, first and second national leagues	1995 to 2021	227 (124 in competition)	80	80	60	[18], [20]
Yüce et al. [20]	Multisport, professional	1970 to 2020	114	42	42	0	[7], [18], [19], [28], [26], [27]
Loturco et al. [29]	Track and field (sprint), training session	Not stated	1	1	1	1	None identified
Teater et al. [28]	American football (NFL)	2018 to 2024 seasons	77	77	77	0	[20] (2018 to 2020 only)
De La Fuente et al. [30]	Track and field (high jump), national competition	Not stated	1	1	1	1	None identified
Petway et al. [26]	Basketball (NBA)	1991 to 2021	27	15	13	13	[7], [20], [27]
Köyağasıoğlu and Lima [27]	Basketball (NBA)	1970 to 2025	27	27	27	17	[7], [20], [26]
Della Villa et al. [31]	Soccer, UEFA Euro 2020	2 July 2021	1	1	1	1	None identified
O'Neill et al. [32]	Multisport, any athletic level	Not stated	495,120 videos screened	57	57	0	Cannot be determined

Periods are the years in which the analyzed ruptures occurred, not the years of publication. Counts are as reported by each study. Overlap is inferred from the sport or league, the period of injury capture, the source database and the stated inclusion criteria, because no included study lists its individual cases, so the extent of overlap cannot be quantified. Della Villa et al. [18] and Hoenig et al. [19] both drew on the same injury database, applied the same restriction to the first and second national divisions and to in-competition injuries, and the period of the former falls entirely within that of the latter, so their samples are likely to overlap substantially. The four studies reporting basketball cases [7,20,26,27] cover overlapping periods within a single league, and the window of Köyağasıoğlu and Lima [27] encompasses those of the other three. Yüce et al. [20] reports 18 basketball, 14 American football and 5 soccer cases and therefore intersects each of the other multi-case cohorts. The eleven publications are best understood as drawing on approximately four distinct athlete populations together with three individually reported cases.

.

Table S5. QA-SIVAS methodological quality appraisal of the eleven included studies.

Study	Total /18	%	Band
Hoenig et al. [19]	16	88.9%	High
De La Fuente et al. [30]	15	83.3%	High
Teater et al. [28]	14	77.8%	Good
Della Villa et al. [18]	14	77.8%	Good
Loturco et al. [29]	14	77.8%	Good
Lemme et al. [7]	13	72.2%	Good
Köyağasıoğlu & Lima [27]	13	72.2%	Good
Petway et al. [26]	12	66.7%	Moderate
Yüce et al. [20]	12	66.7%	Moderate
Della Villa et al. [31]	11	61.1%	Moderate
O’Neill et al. [32]	8	44.4%	Low
**Mean (n = 11)**	**12.9**	**71.7%**	—

Median 13/18 (72.2%); range 8–16 (44.4–88.9%). Bands: low <60%, moderate 60–70%, good 71–80%, high 81–100%.

Table S5. (continued). Item level appraisal matrix (1 = met, green; 0 = not met, red).

Item	[7]	[18]	[19]	[20]	[29]	[28]	[30]	[26]	[27]	[31]	[32]	Met /11
1. Objective	1	1	1	1	1	1	1	1	1	1	1	**11**
2. Sample information	1	1	1	1	0	1	0	1	1	0	1	**8**
3. Sample characteristics	1	1	1	1	1	1	1	1	1	0	0	**9**
4. Video source & quality	0	0	1	0	0	0	1	0	0	1	0	**3**
5. Applied methods	1	1	1	1	1	1	1	1	1	1	1	**11**
6. Systematic approach	1	1	1	1	1	1	1	1	1	1	1	**11**
7. Medical information	0	0	0	0	1	1	1	0	0	0	0	**3**
8. Rater expertise	0	1	1	0	0	0	0	0	0	0	0	**2**
9. Number of raters	1	1	1	1	0	1	0	1	1	0	1	**8**
10. Control group	0	0	0	0	1	0	1	0	0	0	0	**2**
11. Quantitative analysis (validated)	0	0	1	0	1	0	1	0	1	1	0	**5**
12. Main results	1	1	1	1	1	1	1	1	1	1	1	**11**
13. Numbers & proportions	1	1	1	1	1	1	1	1	1	1	1	**11**
14. Injury context	1	1	1	1	1	1	1	1	1	1	1	**11**
15. Example screenshot	1	1	1	0	1	1	1	0	0	1	0	**7**
16. Discussion	1	1	1	1	1	1	1	1	1	1	0	**10**

Item	[7]	[18]	[19]	[20]	[29]	[28]	[30]	[26]	[27]	[31]	[32]	Met /11
17. Clinical implications	1	1	1	1	1	1	1	1	1	1	0	**10**
18. Limitations	1	1	1	1	1	1	1	1	1	0	0	**9**
**Total /18**	**13**	**14**	**16**	**12**	**14**	**14**	**15**	**12**	**13**	**11**	**8**	**142**
**Band**	**Good**	**Good**	**High**	**Mod**	**Good**	**Good**	**High**	**Mod**	**Good**	**Mod**	**Low**	**—**

Item 11 scored 1 only where quantitative kinematics carry documented validity and reliability. Gray shading marks the Met /11 column of per-item counts; the header row and the Total /18 and Band rows are shaded for readability.
